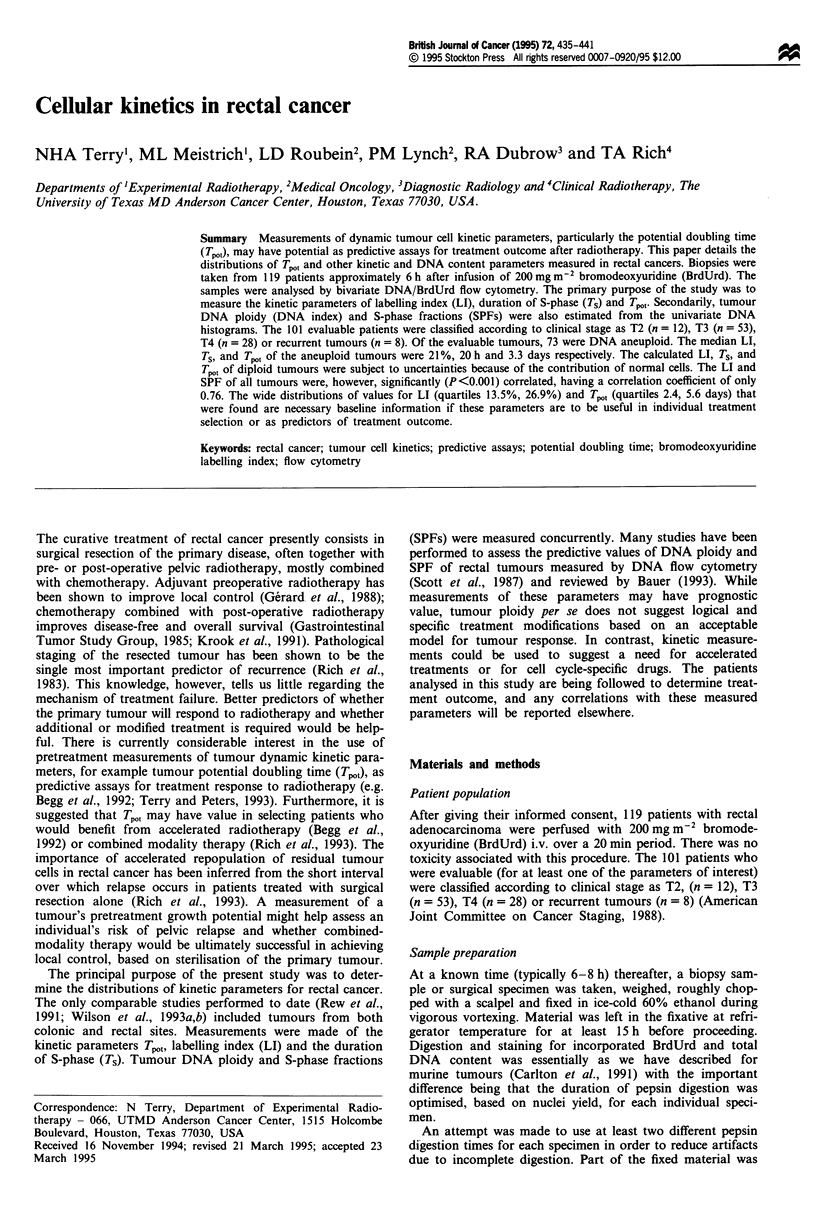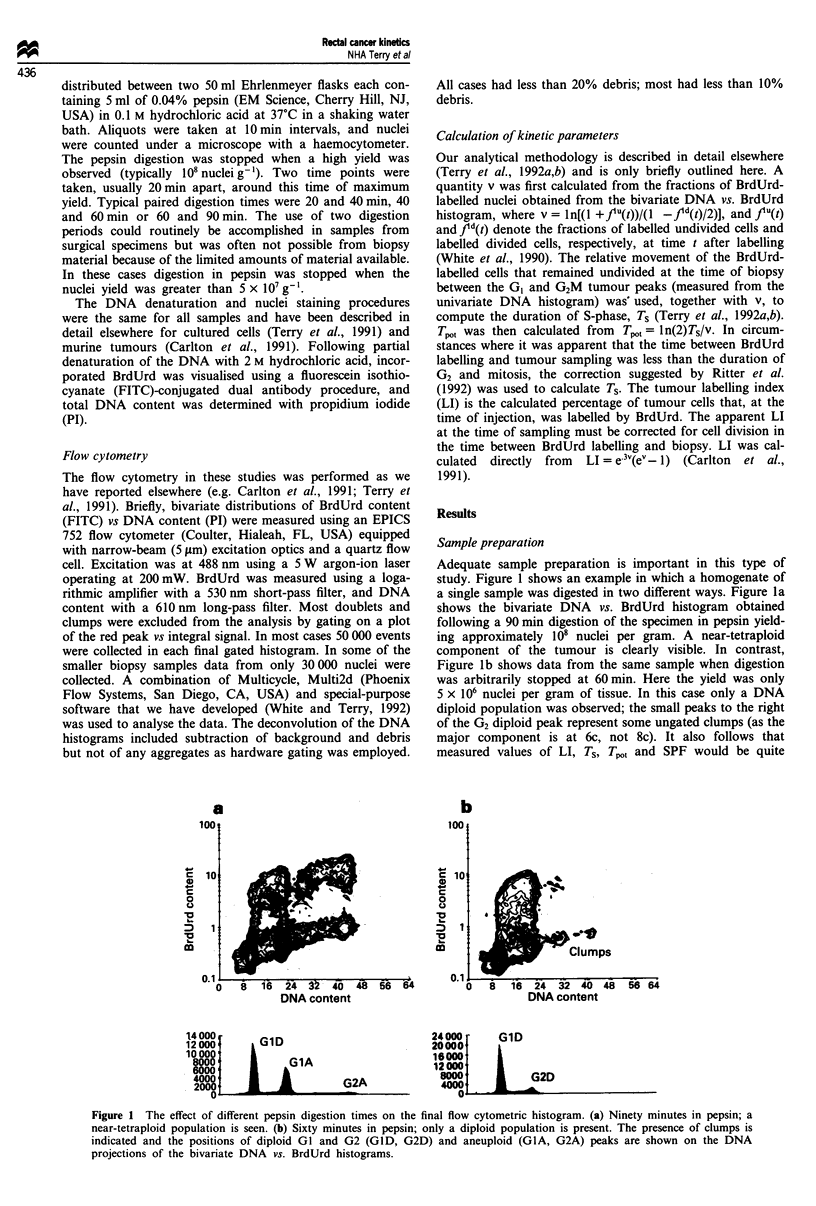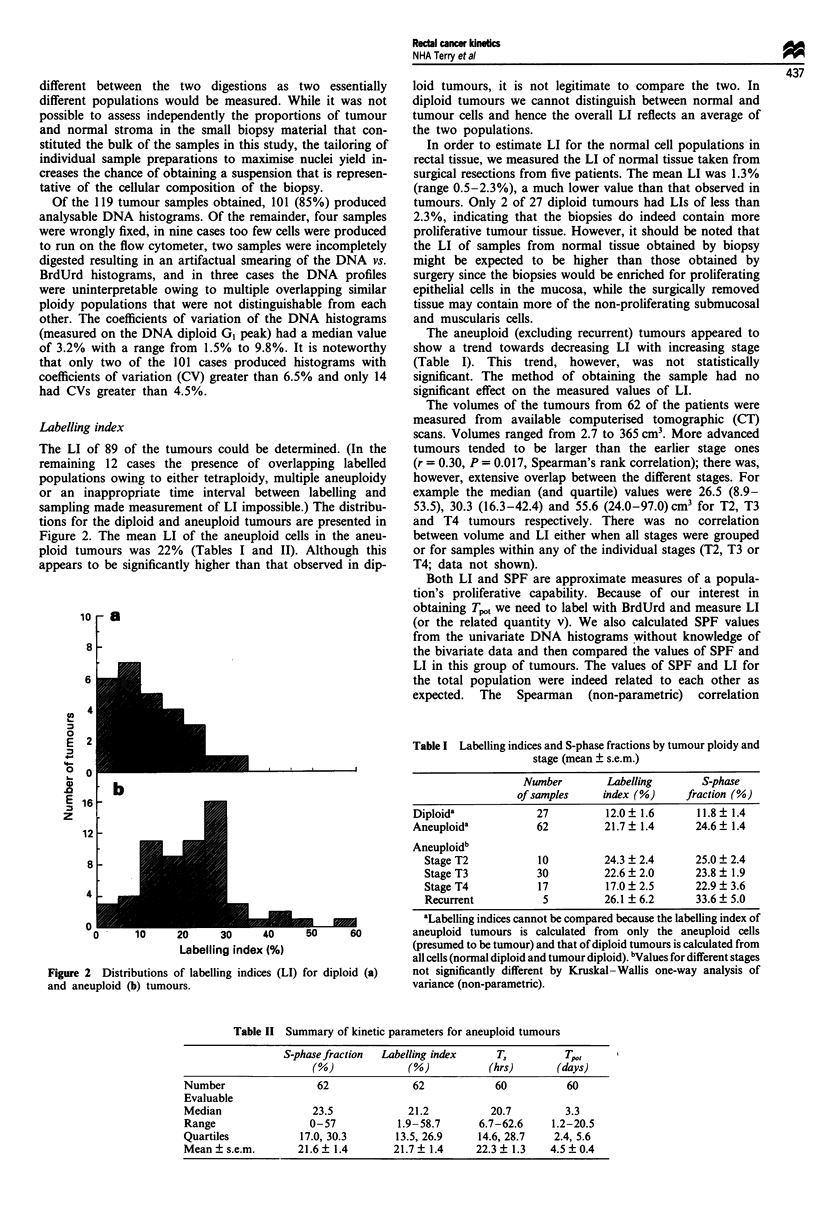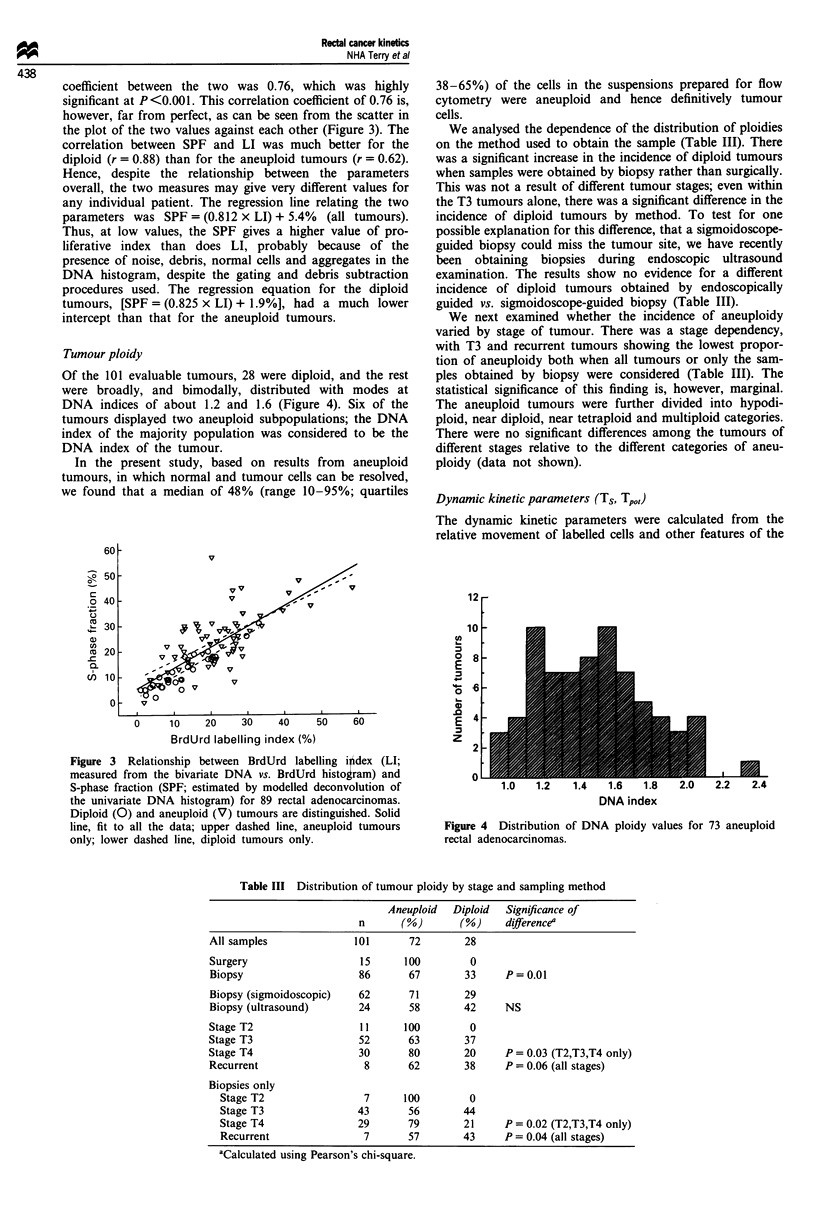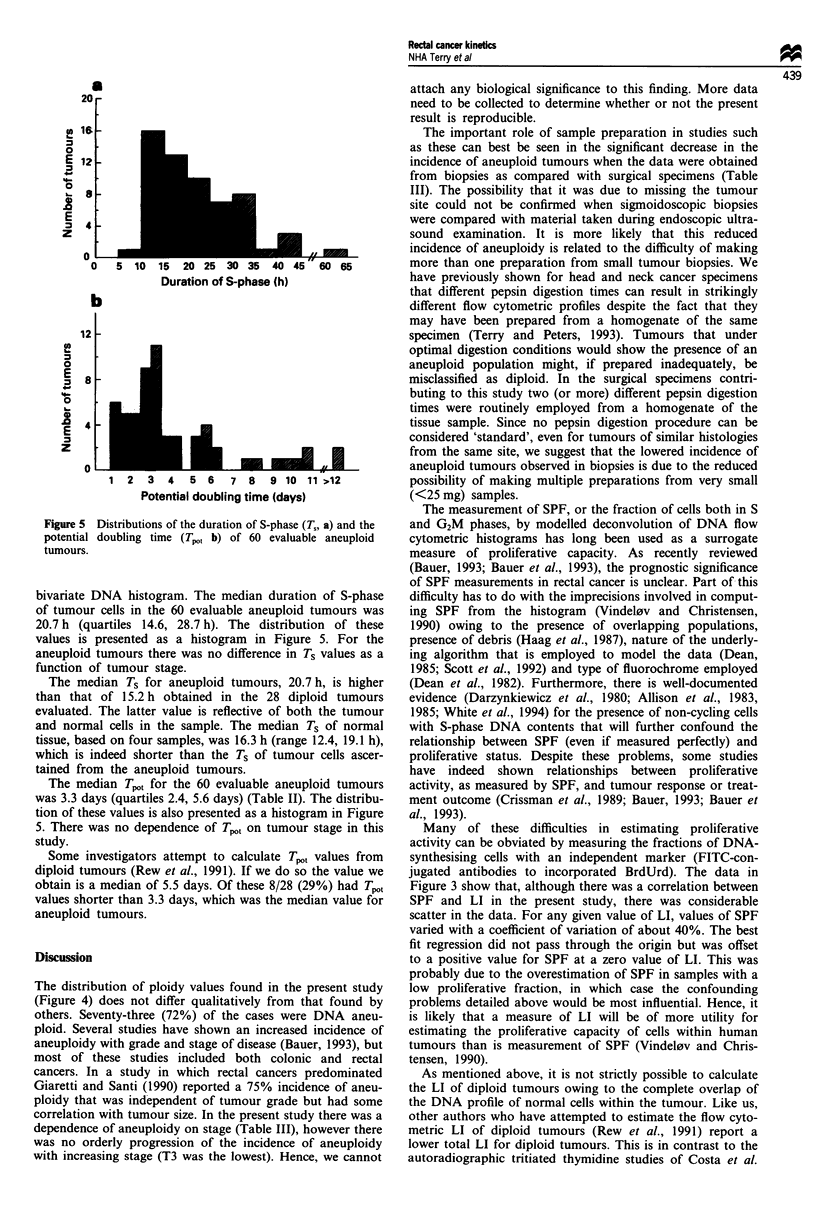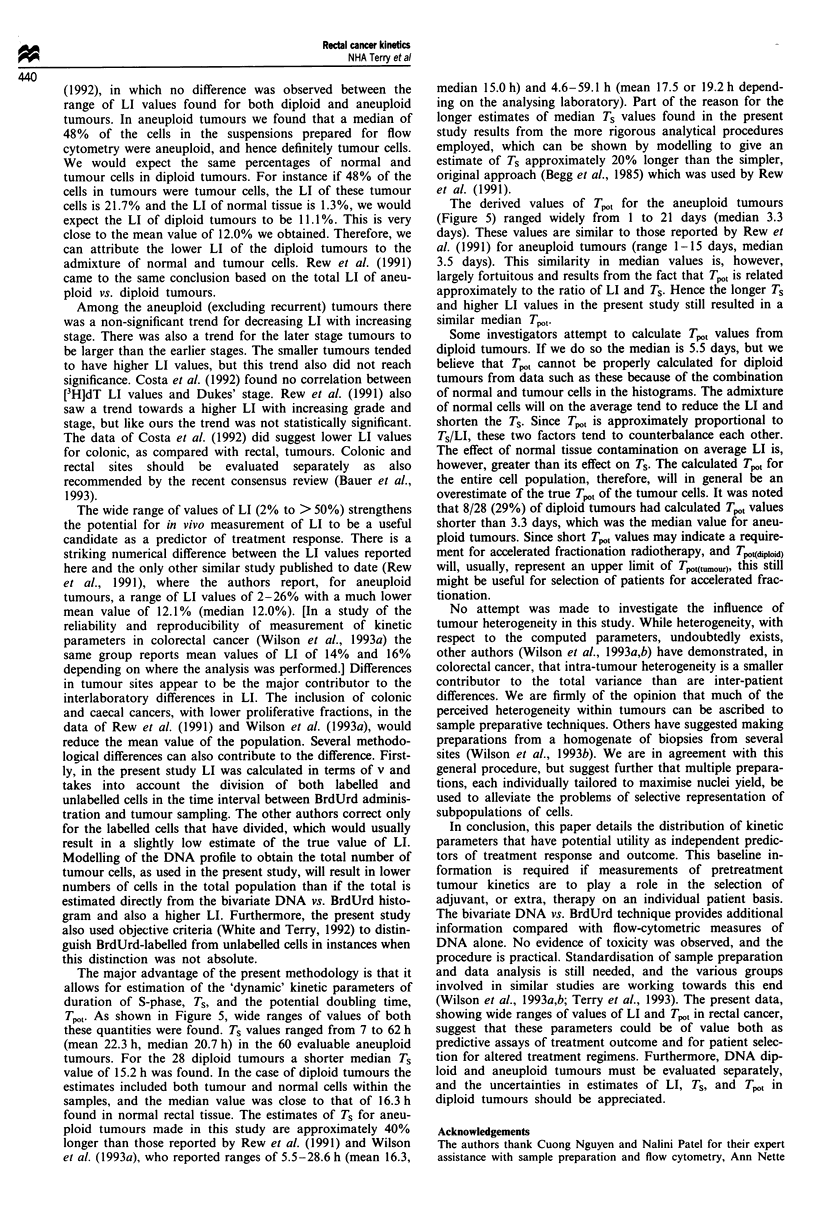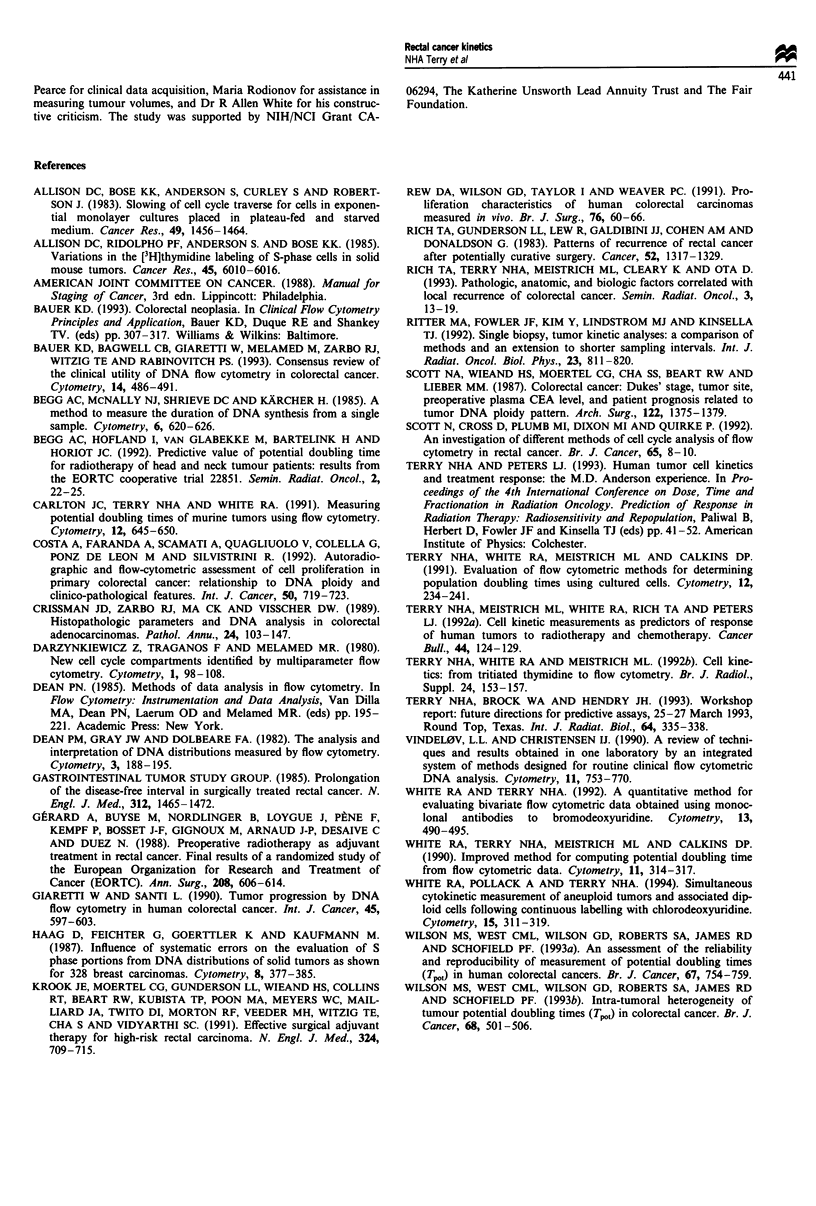# Cellular kinetics in rectal cancer.

**DOI:** 10.1038/bjc.1995.351

**Published:** 1995-08

**Authors:** N. H. Terry, M. L. Meistrich, L. D. Roubein, P. M. Lynch, R. A. Dubrow, T. A. Rich

**Affiliations:** Department of Experimental Radiotherapy, University of Texas MD Anderson Cancer Center, Houston 77030, USA.

## Abstract

Measurements of dynamic tumour cell kinetic parameters, particularly the potential doubling time (Tpot) may have potential as predictive assays for treatment outcome after radiotherapy. This paper details the distributions of Tpot and other kinetic and DNA content parameters measured in rectal cancers. Biopsies were taken from 119 patients approximately 6 h after infusion of 200 mg m-2 bromodeoxyuridine (BrdUrd). The samples were analysed by bivariate DNA/BrdUrd flow cytometry. The primary purpose of the study was to measure the kinetic parameters of labelling index (LI), duration of S-phase (TS) and Tpot. Secondarily, tumour DNA ploidy (DNA index) and S-phase fractions (SPFs) were also estimated from the univariate DNA histograms. The 101 evaluable patients were classified according to clinical stage as T2 (n = 12), T3 (n = 53), T4 (n = 28) or recurrent tumours (n = 8). Of the evaluable tumours, 73 were DNA aneuploid. The median LI, TS, and Tpot of the aneuploid tumours were 21%, 20 h and 3.3 days respectively. The calculated LI, TS, and Tpot of diploid tumours were subject to uncertainties because of the contribution of normal cells. The LI and SPF of all tumours were, however, significantly (P < 0.001) correlated, having a correlation coefficient of only 0.76. The wide distributions of values for LI (quartiles 13.5%, 26.9%) and Tpot (quartiles 2.4, 5.6 days) that were found are necessary baseline information if these parameters are to be useful in individual treatment selection or as predictors of treatment outcome.


British Journal of Cancer (1995) 72, 435–441
© 1995 Stockton Press All rights reserved 0007–0920/95 $12.00

### Cellular kinetics in rectal cancer

NHA Terry[1], ML Meistrich[1], LD Roubein[2], PM Lynch[2], RA Dubrow[3] and TA Rich[4]

*Departments of [1]Experimental Radiotherapy, [2]Medical Oncology, [3]Diagnostic Radiology and [4]Clinical Radiotherapy, The University of Texas MD Anderson Cancer Center, Houston, Texas 77030, USA.*

**Summary** Measurements of dynamic tumour cell kinetic parameters, particularly the potential doubling time ($T_{pot}$), may have potential as predictive assays for treatment outcome after radiotherapy. This paper details the distributions of $T_{pot}$ and other kinetic and DNA content parameters measured in rectal cancers. Biopsies were taken from 119 patients approximately 6 h after infusion of 200 mg $m^{-2}$ bromodeoxyuridine (BrdUrd). The samples were analysed by bivariate DNA/BrdUrd flow cytometry. The primary purpose of the study was to measure the kinetic parameters of labelling index (LI), duration of S-phase ($T_S$) and $T_{pot}$. Secondarily, tumour DNA ploidy (DNA index) and S-phase fractions (SPFs) were also estimated from the univariate DNA histograms. The 101 evaluable patients were classified according to clinical stage as T2 ($n = 12$), T3 ($n = 53$), T4 ($n = 28$) or recurrent tumours ($n = 8$). Of the evaluable tumours, 73 were DNA aneuploid. The median LI, $T_S$, and $T_{pot}$ of the aneuploid tumours were 21%, 20 h and 3.3 days respectively. The calculated LI, $T_S$, and $T_{pot}$ of diploid tumours were subject to uncertainties because of the contribution of normal cells. The LI and SPF of all tumours were, however, significantly ($P < 0.001$) correlated, having a correlation coefficient of only 0.76. The wide distributions of values for LI (quartiles 13.5%, 26.9%) and $T_{pot}$ (quartiles 2.4, 5.6 days) that were found are necessary baseline information if these parameters are to be useful in individual treatment selection or as predictors of treatment outcome.

**Keywords:** rectal cancer; tumour cell kinetics; predictive assays; potential doubling time; bromodeoxyuridine labelling index; flow cytometry

The curative treatment of rectal cancer presently consists in surgical resection of the primary disease, often together with pre- or post-operative pelvic radiotherapy, mostly combined with chemotherapy. Adjuvant preoperative radiotherapy has been shown to improve local control (Gérard *et al.*, 1988); chemotherapy combined with post-operative radiotherapy improves disease-free and overall survival (Gastrointestinal Tumor Study Group, 1985; Krook *et al.*, 1991). Pathological staging of the resected tumour has been shown to be the single most important predictor of recurrence (Rich *et al.*, 1983). This knowledge, however, tells us little regarding the mechanism of treatment failure. Better predictors of whether the primary tumour will respond to radiotherapy and whether additional or modified treatment is required would be helpful. There is currently considerable interest in the use of pretreatment measurements of tumour dynamic kinetic parameters, for example tumour potential doubling time ($T_{pot}$), as predictive assays for treatment response to radiotherapy (e.g. Begg *et al.*, 1992; Terry and Peters, 1993). Furthermore, it is suggested that $T_{pot}$ may have value in selecting patients who would benefit from accelerated radiotherapy (Begg *et al.*, 1992) or combined modality therapy (Rich *et al.*, 1993). The importance of accelerated repopulation of residual tumour cells in rectal cancer has been inferred from the short interval over which relapse occurs in patients treated with surgical resection alone (Rich *et al.*, 1993). A measurement of a tumour's pretreatment growth potential might help assess an individual's risk of pelvic relapse and whether combined-modality therapy would be ultimately successful in achieving local control, based on sterilisation of the primary tumour.

The principal purpose of the present study was to determine the distributions of kinetic parameters for rectal cancer. The only comparable studies performed to date (Rew *et al.*, 1991; Wilson *et al.*, 1993*a*,*b*) included tumours from both colonic and rectal sites. Measurements were made of the kinetic parameters $T_{pot}$, labelling index (LI) and the duration of S-phase ($T_S$). Tumour DNA ploidy and S-phase fractions (SPFs) were measured concurrently. Many studies have been performed to assess the predictive values of DNA ploidy and SPF of rectal tumours measured by DNA flow cytometry (Scott *et al.*, 1987) and reviewed by Bauer (1993). While measurements of these parameters may have prognostic value, tumour ploidy *per se* does not suggest logical and specific treatment modifications based on an acceptable model for tumour response. In contrast, kinetic measurements could be used to suggest a need for accelerated treatments or for cell cycle-specific drugs. The patients analysed in this study are being followed to determine treatment outcome, and any correlations with these measured parameters will be reported elsewhere.

Correspondence: N Terry, Department of Experimental Radiotherapy – 066, UTMD Anderson Cancer Center, 1515 Holcombe Boulevard, Houston, Texas 77030, USA
Received 16 November 1994; revised 21 March 1995; accepted 23 March 1995

### Materials and methods

### *Patient population*

After giving their informed consent, 119 patients with rectal adenocarcinoma were perfused with 200 mg $m^{-2}$ bromodeoxyuridine (BrdUrd) i.v. over a 20 min period. There was no toxicity associated with this procedure. The 101 patients who were evaluable (for at least one of the parameters of interest) were classified according to clinical stage as T2, ($n = 12$), T3 ($n = 53$), T4 ($n = 28$) or recurrent tumours ($n = 8$) (American Joint Committee on Cancer Staging, 1988).

### *Sample preparation*

At a known time (typically 6–8 h) thereafter, a biopsy sample or surgical specimen was taken, weighed, roughly chopped with a scalpel and fixed in ice-cold 60% ethanol during vigorous vortexing. Material was left in the fixative at refrigerator temperature for at least 15 h before proceeding. Digestion and staining for incorporated BrdUrd and total DNA content was essentially as we have described for murine tumours (Carlton *et al.*, 1991) with the important difference being that the duration of pepsin digestion was optimised, based on nuclei yield, for each individual specimen.

An attempt was made to use at least two different pepsin digestion times for each specimen in order to reduce artifacts due to incomplete digestion. Part of the fixed material was distributed between two 50 ml Ehrlenmeyer flasks each containing 5 ml of 0.04% pepsin (EM Science, Cherry Hill, NJ, USA) in 0.1 M hydrochloric acid at 37°C in a shaking water bath. Aliquots were taken at 10 min intervals, and nuclei were counted under a microscope with a haemocytometer. The pepsin digestion was stopped when a high yield was observed (typically $10^8$ nuclei $g^{-1}$). Two time points were taken, usually 20 min apart, around this time of maximum yield. Typical paired digestion times were 20 and 40 min, 40 and 60 min or 60 and 90 min. The use of two digestion periods could routinely be accomplished in samples from surgical specimens but was often not possible from biopsy material because of the limited amounts of material available. In these cases digestion in pepsin was stopped when the nuclei yield was greater than $5 \times 10^7$ $g^{-1}$.

The DNA denaturation and nuclei staining procedures were the same for all samples and have been described in detail elsewhere for cultured cells (Terry *et al.*, 1991) and murine tumours (Carlton *et al.*, 1991). Following partial denaturation of the DNA with 2 M hydrochloric acid, incorporated BrdUrd was visualised using a fluorescein isothiocyanate (FITC)-conjugated dual antibody procedure, and total DNA content was determined with propidium iodide (PI).

### *Flow cytometry*

The flow cytometry in these studies was performed as we have reported elsewhere (e.g. Carlton *et al.*, 1991; Terry *et al.*, 1991). Briefly, bivariate distributions of BrdUrd content (FITC) *vs* DNA content (PI) were measured using an EPICS 752 flow cytometer (Coulter, Hialeah, FL, USA) equipped with narrow-beam (5 μm) excitation optics and a quartz flow cell. Excitation was at 488 nm using a 5 W argon-ion laser operating at 200 mW. BrdUrd was measured using a logarithmic amplifier with a 530 nm short-pass filter, and DNA content with a 610 nm long-pass filter. Most doublets and clumps were excluded from the analysis by gating on a plot of the red peak *vs* integral signal. In most cases 50 000 events were collected in each final gated histogram. In some of the smaller biopsy samples data from only 30 000 nuclei were collected. A combination of Multicycle, Multi2d (Phoenix Flow Systems, San Diego, CA, USA) and special-purpose software that we have developed (White and Terry, 1992) was used to analyse the data. The deconvolution of the DNA histograms included subtraction of background and debris but not of any aggregates as hardware gating was employed. All cases had less than 20% debris; most had less than 10% debris.

### *Calculation of kinetic parameters*

Our analytical methodology is described in detail elsewhere (Terry *et al.*, 1992*a*,*b*) and is only briefly outlined here. A quantity ν was first calculated from the fractions of BrdUrd-labelled nuclei obtained from the bivariate DNA *vs*. BrdUrd histogram, where $\nu = \ln[(1 + f^{\mathrm{lu}}(t))/(1 - f^{\mathrm{ld}}(t)/2)]$, and $f^{\mathrm{lu}}(t)$ and $f^{\mathrm{ld}}(t)$ denote the fractions of labelled undivided cells and labelled divided cells, respectively, at time $t$ after labelling (White *et al.*, 1990). The relative movement of the BrdUrd-labelled cells that remained undivided at the time of biopsy between the $G_1$ and $G_2M$ tumour peaks (measured from the univariate DNA histogram) was' used, together with ν, to compute the duration of S-phase, $T_S$ (Terry *et al.*, 1992*a*,*b*). $T_{pot}$ was then calculated from $T_{pot} = \ln(2)T_S/\nu$. In circumstances where it was apparent that the time between BrdUrd labelling and tumour sampling was less than the duration of $G_2$ and mitosis, the correction suggested by Ritter *et al.* (1992) was used to calculate $T_S$. The tumour labelling index (LI) is the calculated percentage of tumour cells that, at the time of injection, was labelled by BrdUrd. The apparent LI at the time of sampling must be corrected for cell division in the time between BrdUrd labelling and biopsy. LI was calculated directly from $LI = e^{-3\nu}(e^{\nu} - 1)$ (Carlton *et al.*, 1991).

### Results

### *Sample preparation*

Adequate sample preparation is important in this type of study. Figure 1 shows an example in which a homogenate of a single sample was digested in two different ways. Figure 1a shows the bivariate DNA *vs*. BrdUrd histogram obtained following a 90 min digestion of the specimen in pepsin yielding approximately $10^8$ nuclei per gram. A near-tetraploid component of the tumour is clearly visible. In contrast, Figure 1b shows data from the same sample when digestion was arbitrarily stopped at 60 min. Here the yield was only $5 \times 10^6$ nuclei per gram of tissue. In this case only a DNA diploid population was observed; the small peaks to the right of the $G_2$ diploid peak represent some ungated clumps (as the major component is at 6c, not 8c). It also follows that measured values of LI, $T_S$, $T_{pot}$ and SPF would be quite

**Figure 1** The effect of different pepsin digestion times on the final flow cytometric histogram. (**a**) Ninety minutes in pepsin; a near-tetraploid population is seen. (**b**) Sixty minutes in pepsin; only a diploid population is present. The presence of clumps is indicated and the positions of diploid G1 and G2 (G1D, G2D) and aneuploid (G1A, G2A) peaks are shown on the DNA projections of the bivariate DNA *vs*. BrdUrd histograms.

different between the two digestions as two essentially different populations would be measured. While it was not possible to assess independently the proportions of tumour and normal stroma in the small biopsy material that constituted the bulk of the samples in this study, the tailoring of individual sample preparations to maximise nuclei yield increases the chance of obtaining a suspension that is representative of the cellular composition of the biopsy.

Of the 119 tumour samples obtained, 101 (85%) produced analysable DNA histograms. Of the remainder, four samples were wrongly fixed, in nine cases too few cells were produced to run on the flow cytometer, two samples were incompletely digested resulting in an artifactual smearing of the DNA *vs.* BrdUrd histograms, and in three cases the DNA profiles were uninterpretable owing to multiple overlapping similar ploidy populations that were not distinguishable from each other. The coefficients of variation of the DNA histograms (measured on the DNA diploid $G_1$ peak) had a median value of 3.2% with a range from 1.5% to 9.8%. It is noteworthy that only two of the 101 cases produced histograms with coefficients of variation (CV) greater than 6.5% and only 14 had CVs greater than 4.5%.

### *Labelling index*

The LI of 89 of the tumours could be determined. (In the remaining 12 cases the presence of overlapping labelled populations owing to either tetraploidy, multiple aneuploidy or an inappropriate time interval between labelling and sampling made measurement of LI impossible.) The distributions for the diploid and aneuploid tumours are presented in Figure 2. The mean LI of the aneuploid cells in the aneuploid tumours was 22% (Tables I and II). Although this appears to be significantly higher than that observed in diploid tumours, it is not legitimate to compare the two. In diploid tumours we cannot distinguish between normal and tumour cells and hence the overall LI reflects an average of the two populations.

**Figure 2** Distributions of labelling indices (LI) for diploid (**a**) and aneuploid (**b**) tumours.

In order to estimate LI for the normal cell populations in rectal tissue, we measured the LI of normal tissue taken from surgical resections from five patients. The mean LI was 1.3% (range 0.5–2.3%), a much lower value than that observed in tumours. Only 2 of 27 diploid tumours had LIs of less than 2.3%, indicating that the biopsies do indeed contain more proliferative tumour tissue. However, it should be noted that the LI of samples from normal tissue obtained by biopsy might be expected to be higher than those obtained by surgery since the biopsies would be enriched for proliferating epithelial cells in the mucosa, while the surgically removed tissue may contain more of the non-proliferating submucosal and muscularis cells.

The aneuploid (excluding recurrent) tumours appeared to show a trend towards decreasing LI with increasing stage (Table I). This trend, however, was not statistically significant. The method of obtaining the sample had no significant effect on the measured values of LI.

The volumes of the tumours from 62 of the patients were measured from available computerised tomographic (CT) scans. Volumes ranged from 2.7 to 365 $cm^3$. More advanced tumours tended to be larger than the earlier stage ones ($r = 0.30$, $P = 0.017$, Spearman's rank correlation); there was, however, extensive overlap between the different stages. For example the median (and quartile) values were 26.5 (8.9–53.5), 30.3 (16.3–42.4) and 55.6 (24.0–97.0) $cm^3$ for T2, T3 and T4 tumours respectively. There was no correlation between volume and LI either when all stages were grouped or for samples within any of the individual stages (T2, T3 or T4; data not shown).

Both LI and SPF are approximate measures of a population's proliferative capability. Because of our interest in obtaining $T_{pot}$ we need to label with BrdUrd and measure LI (or the related quantity v). We also calculated SPF values from the univariate DNA histograms without knowledge of the bivariate data and then compared the values of SPF and LI in this group of tumours. The values of SPF and LI for the total population were indeed related to each other as expected. The Spearman (non-parametric) correlation

**Table I** Labelling indices and S-phase fractions by tumour ploidy and stage (mean ± s.e.m.)

| | *Number of samples* | *Labelling index (%)* | *S-phase fraction (%)* |
|---|---|---|---|
| Diploid[a] | 27 | 12.0 ± 1.6 | 11.8 ± 1.4 |
| Aneuploid[a] | 62 | 21.7 ± 1.4 | 24.6 ± 1.4 |
| Aneuploid[b] | | | |
| Stage T2 | 10 | 24.3 ± 2.4 | 25.0 ± 2.4 |
| Stage T3 | 30 | 22.6 ± 2.0 | 23.8 ± 1.9 |
| Stage T4 | 17 | 17.0 ± 2.5 | 22.9 ± 3.6 |
| Recurrent | 5 | 26.1 ± 6.2 | 33.6 ± 5.0 |

[a]Labelling indices cannot be compared because the labelling index of aneuploid tumours is calculated from only the aneuploid cells (presumed to be tumour) and that of diploid tumours is calculated from all cells (normal diploid and tumour diploid). [b]Values for different stages not significantly different by Kruskal–Wallis one-way analysis of variance (non-parametric).

**Table II** Summary of kinetic parameters for aneuploid tumours

| | *S-phase fraction (%)* | *Labelling index (%)* | $T_s$ *(hrs)* | $T_{pot}$ *(days)* |
|---|---|---|---|---|
| Number Evaluable | 62 | 62 | 60 | 60 |
| Median | 23.5 | 21.2 | 20.7 | 3.3 |
| Range | 0–57 | 1.9–58.7 | 6.7–62.6 | 1.2–20.5 |
| Quartiles | 17.0, 30.3 | 13.5, 26.9 | 14.6, 28.7 | 2.4, 5.6 |
| Mean ± s.e.m. | 21.6 ± 1.4 | 21.7 ± 1.4 | 22.3 ± 1.3 | 4.5 ± 0.4 |

coefficient between the two was 0.76, which was highly significant at $P<0.001$. This correlation coefficient of 0.76 is, however, far from perfect, as can be seen from the scatter in the plot of the two values against each other (Figure 3). The correlation between SPF and LI was much better for the diploid ($r = 0.88$) than for the aneuploid tumours ($r = 0.62$). Hence, despite the relationship between the parameters overall, the two measures may give very different values for any individual patient. The regression line relating the two parameters was SPF = (0.812 × LI) + 5.4% (all tumours). Thus, at low values, the SPF gives a higher value of proliferative index than does LI, probably because of the presence of noise, debris, normal cells and aggregates in the DNA histogram, despite the gating and debris subtraction procedures used. The regression equation for the diploid tumours, [SPF = (0.825 × LI) + 1.9%], had a much lower intercept than that for the aneuploid tumours.

*Tumour ploidy*

Of the 101 evaluable tumours, 28 were diploid, and the rest were broadly, and bimodally, distributed with modes at DNA indices of about 1.2 and 1.6 (Figure 4). Six of the tumours displayed two aneuploid subpopulations; the DNA index of the majority population was considered to be the DNA index of the tumour.

In the present study, based on results from aneuploid tumours, in which normal and tumour cells can be resolved, we found that a median of 48% (range 10–95%; quartiles 38–65%) of the cells in the suspensions prepared for flow cytometry were aneuploid and hence definitively tumour cells.

We analysed the dependence of the distribution of ploidies on the method used to obtain the sample (Table III). There was a significant increase in the incidence of diploid tumours when samples were obtained by biopsy rather than surgically. This was not a result of different tumour stages; even within the T3 tumours alone, there was a significant difference in the incidence of diploid tumours by method. To test for one possible explanation for this difference, that a sigmoidoscope-guided biopsy could miss the tumour site, we have recently been obtaining biopsies during endoscopic ultrasound examination. The results show no evidence for a different incidence of diploid tumours obtained by endoscopically guided *vs.* sigmoidoscope-guided biopsy (Table III).

We next examined whether the incidence of aneuploidy varied by stage of tumour. There was a stage dependency, with T3 and recurrent tumours showing the lowest proportion of aneuploidy both when all tumours or only the samples obtained by biopsy were considered (Table III). The statistical significance of this finding is, however, marginal. The aneuploid tumours were further divided into hypodiploid, near diploid, near tetraploid and multiploid categories. There were no significant differences among the tumours of different stages relative to the different categories of aneuploidy (data not shown).

*Dynamic kinetic parameters ($T_S$, $T_{pot}$)*

The dynamic kinetic parameters were calculated from the relative movement of labelled cells and other features of the

**Figure 3** Relationship between BrdUrd labelling index (LI; measured from the bivariate DNA *vs.* BrdUrd histogram) and S-phase fraction (SPF; estimated by modelled deconvolution of the univariate DNA histogram) for 89 rectal adenocarcinomas. Diploid (O) and aneuploid (∇) tumours are distinguished. Solid line, fit to all the data; upper dashed line, aneuploid tumours only; lower dashed line, diploid tumours only.

**Figure 4** Distribution of DNA ploidy values for 73 aneuploid rectal adenocarcinomas.

**Table III** Distribution of tumour ploidy by stage and sampling method

| | n | *Aneuploid (%)* | *Diploid (%)* | *Significance of difference*[a] |
|---|---|---|---|---|
| All samples | 101 | 72 | 28 | |
| Surgery | 15 | 100 | 0 | |
| Biopsy | 86 | 67 | 33 | $P = 0.01$ |
| Biopsy (sigmoidoscopic) | 62 | 71 | 29 | |
| Biopsy (ultrasound) | 24 | 58 | 42 | NS |
| Stage T2 | 11 | 100 | 0 | |
| Stage T3 | 52 | 63 | 37 | |
| Stage T4 | 30 | 80 | 20 | $P = 0.03$ (T2,T3,T4 only) |
| Recurrent | 8 | 62 | 38 | $P = 0.06$ (all stages) |
| Biopsies only | | | | |
| Stage T2 | 7 | 100 | 0 | |
| Stage T3 | 43 | 56 | 44 | |
| Stage T4 | 29 | 79 | 21 | $P = 0.02$ (T2,T3,T4 only) |
| Recurrent | 7 | 57 | 43 | $P = 0.04$ (all stages) |

[a]Calculated using Pearson's chi-square.

**Figure 5** Distributions of the duration of S-phase ($T_S$, **a**) and the potential doubling time ($T_{pot}$ **b**) of 60 evaluable aneuploid tumours.

bivariate DNA histogram. The median duration of S-phase of tumour cells in the 60 evaluable aneuploid tumours was 20.7 h (quartiles 14.6, 28.7 h). The distribution of these values is presented as a histogram in Figure 5. For the aneuploid tumours there was no difference in $T_S$ values as a function of tumour stage.

The median $T_S$ for aneuploid tumours, 20.7 h, is higher than that of 15.2 h obtained in the 28 diploid tumours evaluated. The latter value is reflective of both the tumour and normal cells in the sample. The median $T_S$ of normal tissue, based on four samples, was 16.3 h (range 12.4, 19.1 h), which is indeed shorter than the $T_S$ of tumour cells ascertained from the aneuploid tumours.

The median $T_{pot}$ for the 60 evaluable aneuploid tumours was 3.3 days (quartiles 2.4, 5.6 days) (Table II). The distribution of these values is also presented as a histogram in Figure 5. There was no dependence of $T_{pot}$ on tumour stage in this study.

Some investigators attempt to calculate $T_{pot}$ values from diploid tumours (Rew *et al.*, 1991). If we do so the value we obtain is a median of 5.5 days. Of these 8/28 (29%) had $T_{pot}$ values shorter than 3.3 days, which was the median value for aneuploid tumours.

### Discussion

The distribution of ploidy values found in the present study (Figure 4) does not differ qualitatively from that found by others. Seventy-three (72%) of the cases were DNA aneuploid. Several studies have shown an increased incidence of aneuploidy with grade and stage of disease (Bauer, 1993), but most of these studies included both colonic and rectal cancers. In a study in which rectal cancers predominated Giaretti and Santi (1990) reported a 75% incidence of aneuploidy that was independent of tumour grade but had some correlation with tumour size. In the present study there was a dependence of aneuploidy on stage (Table III), however there was no orderly progression of the incidence of aneuploidy with increasing stage (T3 was the lowest). Hence, we cannot attach any biological significance to this finding. More data need to be collected to determine whether or not the present result is reproducible.

The important role of sample preparation in studies such as these can best be seen in the significant decrease in the incidence of aneuploid tumours when the data were obtained from biopsies as compared with surgical specimens (Table III). The possibility that it was due to missing the tumour site could not be confirmed when sigmoidoscopic biopsies were compared with material taken during endoscopic ultrasound examination. It is more likely that this reduced incidence of aneuploidy is related to the difficulty of making more than one preparation from small tumour biopsies. We have previously shown for head and neck cancer specimens that different pepsin digestion times can result in strikingly different flow cytometric profiles despite the fact that they may have been prepared from a homogenate of the same specimen (Terry and Peters, 1993). Tumours that under optimal digestion conditions would show the presence of an aneuploid population might, if prepared inadequately, be misclassified as diploid. In the surgical specimens contributing to this study two (or more) different pepsin digestion times were routinely employed from a homogenate of the tissue sample. Since no pepsin digestion procedure can be considered 'standard', even for tumours of similar histologies from the same site, we suggest that the lowered incidence of aneuploid tumours observed in biopsies is due to the reduced possibility of making multiple preparations from very small (<25 mg) samples.

The measurement of SPF, or the fraction of cells both in S and $G_2M$ phases, by modelled deconvolution of DNA flow cytometric histograms has long been used as a surrogate measure of proliferative capacity. As recently reviewed (Bauer, 1993; Bauer *et al.*, 1993), the prognostic significance of SPF measurements in rectal cancer is unclear. Part of this difficulty has to do with the imprecisions involved in computing SPF from the histogram (Vindeløv and Christensen, 1990) owing to the presence of overlapping populations, presence of debris (Haag *et al.*, 1987), nature of the underlying algorithm that is employed to model the data (Dean, 1985; Scott *et al.*, 1992) and type of fluorochrome employed (Dean *et al.*, 1982). Furthermore, there is well-documented evidence (Darzynkiewicz *et al.*, 1980; Allison *et al.*, 1983, 1985; White *et al.*, 1994) for the presence of non-cycling cells with S-phase DNA contents that will further confound the relationship between SPF (even if measured perfectly) and proliferative status. Despite these problems, some studies have indeed shown relationships between proliferative activity, as measured by SPF, and tumour response or treatment outcome (Crissman *et al.*, 1989; Bauer, 1993; Bauer *et al.*, 1993).

Many of these difficulties in estimating proliferative activity can be obviated by measuring the fractions of DNA-synthesising cells with an independent marker (FITC-conjugated antibodies to incorporated BrdUrd). The data in Figure 3 show that, although there was a correlation between SPF and LI in the present study, there was considerable scatter in the data. For any given value of LI, values of SPF varied with a coefficient of variation of about 40%. The best fit regression did not pass through the origin but was offset to a positive value for SPF at a zero value of LI. This was probably due to the overestimation of SPF in samples with a low proliferative fraction, in which case the confounding problems detailed above would be most influential. Hence, it is likely that a measure of LI will be of more utility for estimating the proliferative capacity of cells within human tumours than is measurement of SPF (Vindeløv and Christensen, 1990).

As mentioned above, it is not strictly possible to calculate the LI of diploid tumours owing to the complete overlap of the DNA profile of normal cells within the tumour. Like us, other authors who have attempted to estimate the flow cytometric LI of diploid tumours (Rew *et al.*, 1991) report a lower total LI for diploid tumours. This is in contrast to the autoradiographic tritiated thymidine studies of Costa *et al.*

(1992), in which no difference was observed between the range of LI values found for both diploid and aneuploid tumours. In aneuploid tumours we found that a median of 48% of the cells in the suspensions prepared for flow cytometry were aneuploid, and hence definitely tumour cells. We would expect the same percentages of normal and tumour cells in diploid tumours. For instance if 48% of the cells in tumours were tumour cells, the LI of these tumour cells is 21.7% and the LI of normal tissue is 1.3%, we would expect the LI of diploid tumours to be 11.1%. This is very close to the mean value of 12.0% we obtained. Therefore, we can attribute the lower LI of the diploid tumours to the admixture of normal and tumour cells. Rew *et al.* (1991) came to the same conclusion based on the total LI of aneuploid *vs.* diploid tumours.

Among the aneuploid (excluding recurrent) tumours there was a non-significant trend for decreasing LI with increasing stage. There was also a trend for the later stage tumours to be larger than the earlier stages. The smaller tumours tended to have higher LI values, but this trend also did not reach significance. Costa *et al.* (1992) found no correlation between $[^3H]$dT LI values and Dukes' stage. Rew *et al.* (1991) also saw a trend towards a higher LI with increasing grade and stage, but like ours the trend was not statistically significant. The data of Costa *et al.* (1992) did suggest lower LI values for colonic, as compared with rectal, tumours. Colonic and rectal sites should be evaluated separately as also recommended by the recent consensus review (Bauer *et al.*, 1993).

The wide range of values of LI (2% to >50%) strengthens the potential for *in vivo* measurement of LI to be a useful candidate as a predictor of treatment response. There is a striking numerical difference between the LI values reported here and the only other similar study published to date (Rew *et al.*, 1991), where the authors report, for aneuploid tumours, a range of LI values of 2–26% with a much lower mean value of 12.1% (median 12.0%). [In a study of the reliability and reproducibility of measurement of kinetic parameters in colorectal cancer (Wilson *et al.*, 1993*a*) the same group reports mean values of LI of 14% and 16% depending on where the analysis was performed.] Differences in tumour sites appear to be the major contributor to the interlaboratory differences in LI. The inclusion of colonic and caecal cancers, with lower proliferative fractions, in the data of Rew *et al.* (1991) and Wilson *et al.* (1993*a*), would reduce the mean value of the population. Several methodological differences can also contribute to the difference. Firstly, in the present study LI was calculated in terms of v and takes into account the division of both labelled and unlabelled cells in the time interval between BrdUrd administration and tumour sampling. The other authors correct only for the labelled cells that have divided, which would usually result in a slightly low estimate of the true value of LI. Modelling of the DNA profile to obtain the total number of tumour cells, as used in the present study, will result in lower numbers of cells in the total population than if the total is estimated directly from the bivariate DNA *vs.* BrdUrd histogram and also a higher LI. Furthermore, the present study also used objective criteria (White and Terry, 1992) to distinguish BrdUrd-labelled from unlabelled cells in instances when this distinction was not absolute.

The major advantage of the present methodology is that it allows for estimation of the 'dynamic' kinetic parameters of duration of S-phase, $T_S$, and the potential doubling time, $T_{pot}$. As shown in Figure 5, wide ranges of values of both these quantities were found. $T_S$ values ranged from 7 to 62 h (mean 22.3 h, median 20.7 h) in the 60 evaluable aneuploid tumours. For the 28 diploid tumours a shorter median $T_S$ value of 15.2 h was found. In the case of diploid tumours the estimates included both tumour and normal cells within the samples, and the median value was close to that of 16.3 h found in normal rectal tissue. The estimates of $T_S$ for aneuploid tumours made in this study are approximately 40% longer than those reported by Rew *et al.* (1991) and Wilson *et al.* (1993*a*), who reported ranges of 5.5–28.6 h (mean 16.3, median 15.0 h) and 4.6–59.1 h (mean 17.5 or 19.2 h depending on the analysing laboratory). Part of the reason for the longer estimates of median $T_S$ values found in the present study results from the more rigorous analytical procedures employed, which can be shown by modelling to give an estimate of $T_S$ approximately 20% longer than the simpler, original approach (Begg *et al.*, 1985) which was used by Rew *et al.* (1991).

The derived values of $T_{pot}$ for the aneuploid tumours (Figure 5) ranged widely from 1 to 21 days (median 3.3 days). These values are similar to those reported by Rew *et al.* (1991) for aneuploid tumours (range 1–15 days, median 3.5 days). This similarity in median values is, however, largely fortuitous and results from the fact that $T_{pot}$ is related approximately to the ratio of LI and $T_S$. Hence the longer $T_S$ and higher LI values in the present study still resulted in a similar median $T_{pot}$.

Some investigators attempt to calculate $T_{pot}$ values from diploid tumours. If we do so the median is 5.5 days, but we believe that $T_{pot}$ cannot be properly calculated for diploid tumours from data such as these because of the combination of normal and tumour cells in the histograms. The admixture of normal cells will on the average tend to reduce the LI and shorten the $T_S$. Since $T_{pot}$ is approximately proportional to $T_S$/LI, these two factors tend to counterbalance each other. The effect of normal tissue contamination on average LI is, however, greater than its effect on $T_S$. The calculated $T_{pot}$ for the entire cell population, therefore, will in general be an overestimate of the true $T_{pot}$ of the tumour cells. It was noted that 8/28 (29%) of diploid tumours had calculated $T_{pot}$ values shorter than 3.3 days, which was the median value for aneuploid tumours. Since short $T_{pot}$ values may indicate a requirement for accelerated fractionation radiotherapy, and $T_{pot(diploid)}$ will, usually, represent an upper limit of $T_{pot(tumour)}$, this still might be useful for selection of patients for accelerated fractionation.

No attempt was made to investigate the influence of tumour heterogeneity in this study. While heterogeneity, with respect to the computed parameters, undoubtedly exists, other authors (Wilson *et al.*, 1993*a*,*b*) have demonstrated, in colorectal cancer, that intra-tumour heterogeneity is a smaller contributor to the total variance than are inter-patient differences. We are firmly of the opinion that much of the perceived heterogeneity within tumours can be ascribed to sample preparative techniques. Others have suggested making preparations from a homogenate of biopsies from several sites (Wilson *et al.*, 1993*b*). We are in agreement with this general procedure, but suggest further that multiple preparations, each individually tailored to maximise nuclei yield, be used to alleviate the problems of selective representation of subpopulations of cells.

In conclusion, this paper details the distribution of kinetic parameters that have potential utility as independent predictors of treatment response and outcome. This baseline information is required if measurements of pretreatment tumour kinetics are to play a role in the selection of adjuvant, or extra, therapy on an individual patient basis. The bivariate DNA *vs.* BrdUrd technique provides additional information compared with flow-cytometric measures of DNA alone. No evidence of toxicity was observed, and the procedure is practical. Standardisation of sample preparation and data analysis is still needed, and the various groups involved in similar studies are working towards this end (Wilson *et al.*, 1993*a*,*b*; Terry *et al.*, 1993). The present data, showing wide ranges of values of LI and $T_{pot}$ in rectal cancer, suggest that these parameters could be of value both as predictive assays of treatment outcome and for patient selection for altered treatment regimens. Furthermore, DNA diploid and aneuploid tumours must be evaluated separately, and the uncertainties in estimates of LI, $T_S$, and $T_{pot}$ in diploid tumours should be appreciated.

**Acknowledgements**
The authors thank Cuong Nguyen and Nalini Patel for their expert assistance with sample preparation and flow cytometry, Ann Nette

Pearce for clinical data acquisition, Maria Rodionov for assistance in measuring tumour volumes, and Dr R Allen White for his constructive criticism. The study was supported by NIH/NCI Grant CA-06294, The Katherine Unsworth Lead Annuity Trust and The Fair Foundation.

### References

ALLISON DC, BOSE KK, ANDERSON S, CURLEY S AND ROBERTSON J. (1983). Slowing of cell cycle traverse for cells in exponential monolayer cultures placed in plateau-fed and starved medium. *Cancer Res.*, **49**, 1456–1464.

ALLISON DC, RIDOLPHO PF, ANDERSON S. AND BOSE KK. (1985). Variations in the [$^3$H]thymidine labeling of S-phase cells in solid mouse tumors. *Cancer Res.*, **45**, 6010–6016.

AMERICAN JOINT COMMITTEE ON CANCER. (1988). *Manual for Staging of Cancer*, 3rd edn. Lippincott: Philadelphia.

BAUER KD. (1993). Colorectal neoplasia. In *Clinical Flow Cytometry Principles and Application*, Bauer KD, Duque RE and Shankey TV. (eds) pp. 307–317. Williams & Wilkins: Baltimore.

BAUER KD, BAGWELL CB, GIARETTI W, MELAMED M, ZARBO RJ, WITZIG TE AND RABINOVITCH PS. (1993). Consensus review of the clinical utility of DNA flow cytometry in colorectal cancer. *Cytometry*, **14**, 486–491.

BEGG AC, MCNALLY NJ, SHRIEVE DC AND KÄRCHER H. (1985). A method to measure the duration of DNA synthesis from a single sample. *Cytometry*, **6**, 620–626.

BEGG AC, HOFLAND I, VAN GLABEKKE M, BARTELINK H AND HORIOT JC. (1992). Predictive value of potential doubling time for radiotherapy of head and neck tumour patients: results from the EORTC cooperative trial 22851. *Semin. Radiat. Oncol.*, **2**, 22–25.

CARLTON JC, TERRY NHA AND WHITE RA. (1991). Measuring potential doubling times of murine tumors using flow cytometry. *Cytometry*, **12**, 645–650.

COSTA A, FARANDA A, SCAMATI A, QUAGLIUOLO V, COLELLA G, PONZ DE LEON M AND SILVISTRINI R. (1992). Autoradiographic and flow-cytometric assessment of cell proliferation in primary colorectal cancer: relationship to DNA ploidy and clinico-pathological features. *Int. J. Cancer*, **50**, 719–723.

CRISSMAN JD, ZARBO RJ, MA CK AND VISSCHER DW. (1989). Histopathologic parameters and DNA analysis in colorectal adenocarcinomas. *Pathol. Annu.*, **24**, 103–147.

DARZYNKIEWICZ Z, TRAGANOS F AND MELAMED MR. (1980). New cell cycle compartments identified by multiparameter flow cytometry. *Cytometry*, **1**, 98–108.

DEAN PN. (1985). Methods of data analysis in flow cytometry. In *Flow Cytometry: Instrumentation and Data Analysis*, Van Dilla MA, Dean PN, Laerum OD and Melamed MR. (eds) pp. 195–221. Academic Press: New York.

DEAN PM, GRAY JW AND DOLBEARE FA. (1982). The analysis and interpretation of DNA distributions measured by flow cytometry. *Cytometry*, **3**, 188–195.

GASTROINTESTINAL TUMOR STUDY GROUP. (1985). Prolongation of the disease-free interval in surgically treated rectal cancer. *N. Engl. J. Med.*, **312**, 1465–1472.

GÉRARD A, BUYSE M, NORDLINGER B, LOYGUE J, PÈNE F, KEMPF P, BOSSET J-F, GIGNOUX M, ARNAUD J-P, DESAIVE C AND DUEZ N. (1988). Preoperative radiotherapy as adjuvant treatment in rectal cancer. Final results of a randomized study of the European Organization for Research and Treatment of Cancer (EORTC). *Ann. Surg.*, **208**, 606–614.

GIARETTI W AND SANTI L. (1990). Tumor progression by DNA flow cytometry in human colorectal cancer. *Int. J. Cancer*, **45**, 597–603.

HAAG D, FEICHTER G, GOERTTLER K AND KAUFMANN M. (1987). Influence of systematic errors on the evaluation of S phase portions from DNA distributions of solid tumors as shown for 328 breast carcinomas. *Cytometry*, **8**, 377–385.

KROOK JE, MOERTEL CG, GUNDERSON LL, WIEAND HS, COLLINS RT, BEART RW, KUBISTA TP, POON MA, MEYERS WC, MAILLIARD JA, TWITO DI, MORTON RF, VEEDER MH, WITZIG TE, CHA S AND VIDYARTHI SC. (1991). Effective surgical adjuvant therapy for high-risk rectal carcinoma. *N. Engl. J. Med.*, **324**, 709–715.

REW DA, WILSON GD, TAYLOR I AND WEAVER PC. (1991). Proliferation characteristics of human colorectal carcinomas measured *in vivo*. *Br. J. Surg.*, **76**, 60–66.

RICH TA, GUNDERSON LL, LEW R, GALDIBINI JJ, COHEN AM AND DONALDSON G. (1983). Patterns of recurrence of rectal cancer after potentially curative surgery. *Cancer*, **52**, 1317–1329.

RICH TA, TERRY NHA, MEISTRICH ML, CLEARY K AND OTA D. (1993). Pathologic, anatomic, and biologic factors correlated with local recurrence of colorectal cancer. *Semin. Radiat. Oncol.*, **3**, 13–19.

RITTER MA, FOWLER JF, KIM Y, LINDSTROM MJ AND KINSELLA TJ. (1992). Single biopsy, tumor kinetic analyses: a comparison of methods and an extension to shorter sampling intervals. *Int. J. Radiat. Oncol. Biol. Phys.*, **23**, 811–820.

SCOTT NA, WIEAND HS, MOERTEL CG, CHA SS, BEART RW AND LIEBER MM. (1987). Colorectal cancer: Dukes' stage, tumor site, preoperative plasma CEA level, and patient prognosis related to tumor DNA ploidy pattern. *Arch. Surg.*, **122**, 1375–1379.

SCOTT N, CROSS D, PLUMB MI, DIXON MI AND QUIRKE P. (1992). An investigation of different methods of cell cycle analysis of flow cytometry in rectal cancer. *Br. J. Cancer*, **65**, 8–10.

TERRY NHA AND PETERS LJ. (1993). Human tumor cell kinetics and treatment response: the M.D. Anderson experience. In *Proceedings of the 4th International Conference on Dose, Time and Fractionation in Radiation Oncology. Prediction of Response in Radiation Therapy: Radiosensitivity and Repopulation*, Paliwal B, Herbert D, Fowler JF and Kinsella TJ (eds) pp. 41–52. American Institute of Physics: Colchester.

TERRY NHA, WHITE RA, MEISTRICH ML AND CALKINS DP. (1991). Evaluation of flow cytometric methods for determining population doubling times using cultured cells. *Cytometry*, **12**, 234–241.

TERRY NHA, MEISTRICH ML, WHITE RA, RICH TA AND PETERS LJ. (1992*a*). Cell kinetic measurements as predictors of response of human tumors to radiotherapy and chemotherapy. *Cancer Bull.*, **44**, 124–129.

TERRY NHA, WHITE RA AND MEISTRICH ML. (1992*b*). Cell kinetics: from tritiated thymidine to flow cytometry. *Br. J. Radiol.*, Suppl. 24, 153–157.

TERRY NHA, BROCK WA AND HENDRY JH. (1993). Workshop report: future directions for predictive assays, 25–27 March 1993, Round Top, Texas. *Int. J. Radiat. Biol.*, **64**, 335–338.

VINDELØV, L.L. AND CHRISTENSEN IJ. (1990). A review of techniques and results obtained in one laboratory by an integrated system of methods designed for routine clinical flow cytometric DNA analysis. *Cytometry*, **11**, 753–770.

WHITE RA AND TERRY NHA. (1992). A quantitative method for evaluating bivariate flow cytometric data obtained using monoclonal antibodies to bromodeoxyuridine. *Cytometry*, **13**, 490–495.

WHITE RA, TERRY NHA, MEISTRICH ML AND CALKINS DP. (1990). Improved method for computing potential doubling time from flow cytometric data. *Cytometry*, **11**, 314–317.

WHITE RA, POLLACK A AND TERRY NHA. (1994). Simultaneous cytokinetic measurement of aneuploid tumors and associated diploid cells following continuous labelling with chlorodeoxyuridine. *Cytometry*, **15**, 311–319.

WILSON MS, WEST CML, WILSON GD, ROBERTS SA, JAMES RD AND SCHOFIELD PF. (1993*a*). An assessment of the reliability and reproducibility of measurement of potential doubling times ($T_{pot}$) in human colorectal cancers. *Br. J. Cancer*, **67**, 754–759.

WILSON MS, WEST CML, WILSON GD, ROBERTS SA, JAMES RD AND SCHOFIELD PF. (1993*b*). Intra-tumoral heterogeneity of tumour potential doubling times ($T_{pot}$) in colorectal cancer. *Br. J. Cancer*, **68**, 501–506.